# Percutaneous Cement-Augmented Screws Fixation in the Fractures of the Aging Spine: Is It the Solution?

**DOI:** 10.1155/2014/610675

**Published:** 2014-02-20

**Authors:** Sébastien Pesenti, Benjamin Blondel, Emilie Peltier, Tarek Adetchessi, Henry Dufour, Stéphane Fuentes

**Affiliations:** ^1^Spine Division, Aix-Marseille University, Hôpital de la Timone, 264 Rue Saint Pierre, 13005 Marseille, France; ^2^Service de Neurochirurgie, Hôpital de la Timone, 264 rue Saint Pierre, 13005 Marseille, France

## Abstract

*Introduction*. Management of elderly patients with thoracolumbar fractures is still challenging due to frequent osteoporosis and risk of screws pull-out. The aim of this study was to evaluate results of a percutaneous-only procedure to treat these fragile patients using cement-augmented screws. *Methods*. 12 patients diagnosed with a thoracolumbar fracture associated with an important loss of bone stock were included in this prospective study. Surgical procedure included systematically a percutaneous osteosynthesis using cemented fenestrated screws. When necessary, additional anterior support was performed using a kyphoplasty procedure. Clinical and radiographic evaluations were performed using CT scan. *Results*. On the whole series, 15 fractures were diagnosed and 96 cemented screws were inserted. The difference between the pre- and postoperative vertebral kyphosis was statistically significant (12.9° versus 4.4°, *P* = 0.0006). No extrapedicular screw was reported and one patient was diagnosed with a cement-related pulmonary embolism. During follow-up period, no infectious complications, implant failures, or pull-out screws were noticed. *Discussion*. Aging spine is becoming an increasing public health issue. Management of these patients requires specific attention due to the augmented risk of complications. Using percutaneous-only screws fixation with cemented screw provides satisfactory results. A rigorous technique is mandatory in order to achieve best outcomes.

## 1. Introduction

Due to the lengthening of life expectancy, spine physicians are more and more likely to deal with age-related changes. Aging spine has been associated to various sagittal changes such as a loss of lumbar lordosis, an increased thoracic kyphosis, and eventually compensatory mechanisms such as pelvic retroversion and knee flexion in order to keep the head over the pelvis [1].

However, one of the biggest challenges in this population is the loss of bone stock that can lead to real osteoporosis, defined by the WHO as a T-score below −2.5. This critical situation is responsible for a high risk of implant failures during spine surgery and of pseudarthrosis.

According to these age-related changes, management of vertebral fractures in elderly remains a challenge. In order to improve safety in implant anchorage and better clinical outcomes, various systems have been developed for osteoporotic bone such as expandable screws and partially or fully cannulated fenestrated screws [2–5]. Among them, cemented screws have been described in the last 20 years and recent developments have increased its safety with in vitro and in vivo reports [6–8].

On the other hand, in the recent years, percutaneous spine surgery has become increasingly popular. The objectives of these minimal invasive procedures are to limit muscular damages, to decrease postoperative pain, to decrease length of stay, and to accelerate postoperative recovery.

The aim of this study was to report our experience in the management of elderly thoracolumbar fractures using percutaneous cemented screws.

## 2. Methods

### 2.1. Study Design

**Figure 1:**
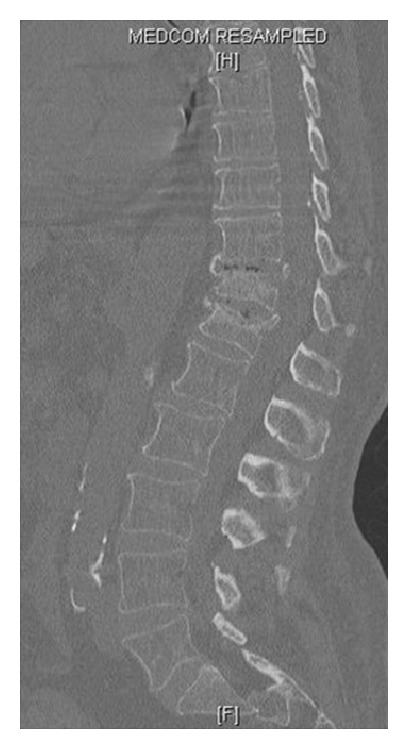
Preoperative sagittal CT scan of a 72-year-old male with T11 and T12 fractures.

Between January 2012 and July 2013, 12 patients have been included in this retrospective study (7 females, 5 males). All of them were admitted in our institution for management of a thoracolumbar fracture. The inclusion criteria were patients ageing over 60 years old, diagnosed with a thoracolumbar fracture requiring a surgical treatment (Figure 1), with severe osteoporosis confirmed by a previous osteodensitometry showing a T-score < −2.5 or low bone density secondary to tumoral or inflammatory disease. Patients younger than 60 years old or without history of poor bone stock were excluded from the study. During postoperative course, each patient was followed up during at least 3 months.

### 2.2. Surgical Procedure

**Figure 2:**
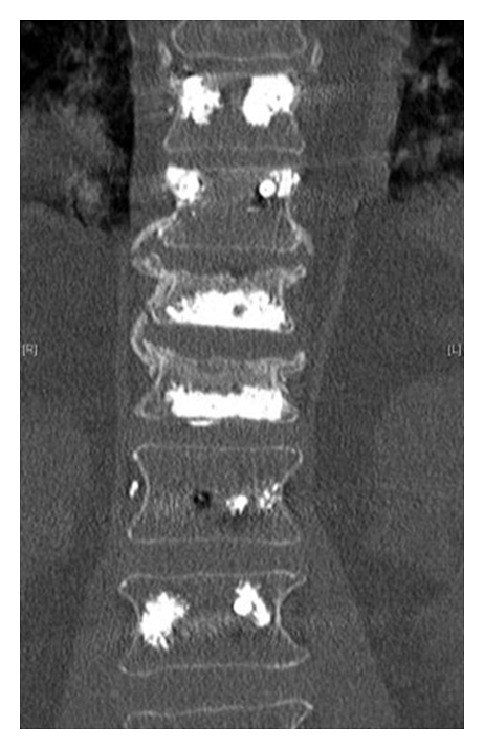
Postoperative coronal CT scan (same patient in Figure 1) showing cemented screw 2 levels above and below the fractured levels and 2 balloon kyphoplasty.

On the whole series, surgical procedure was standardized and performed by a single senior surgeon of our department. In every case, fixation of the fracture was done using a posterior percutaneous transpedicular instrumentation using cement-augmented cannulated fenestrated screws (Longitude, Medtronic, or Mantis, Stryker). Screws used in this study were partially cannulated fenestrated screws that allow injection of the cement in the first third of the screw when compared to fully cannulated fenestrated screws [9]. Pedicle screws were systematically inserted under anteroposterior and lateral fluoroscopic guidance. Screws diameter was 5.5 or 6.5 mm, depending on the level of the fracture, and length was determined based on the preoperative CT scan. Once inserted, on each screw, approximatively 1.8 mL (1.5–2.5 mL) of PMMA cement (Kypho, Medtronic) was then injected into the vertebral bodies through the pedicular screws under fluoroscopic control to prevent cement leakage. Finally, two rods were contoured according to sagittal alignment of the patient and inserted percutaneously in order to restore vertebral body height loss and traumatic kyphosis. When needed, an anterior support of the vertebral body was performed using a balloon kyphoplasty on the fractured level (Figure 2).

### 2.3. Radiologic Evaluation

Pre- and postoperative low-dose CT scans were systematically obtained. The following measurements were performed in order to evaluate the correction obtained after the surgical procedure: vertebral and local kyphosis and Beck's index (defined as the ratio between the height of the anterior wall and the posterior of the fractured vertebral body). Of note, radiologic evaluation of the kyphosis reduction is not only due to the use of cemented screws that allows a better distraction maneuver but also mostly related to the balloon kyphoplasty that was realized in all cases but in one. Objective of the radiologic evaluation was to report results of deformity correction using this percutaneous-only technique.

### 2.4. Clinical Evaluation

For each patient, clinical outcomes were evaluated using demographic data, length of stay, pain medications pre- and postoperatively, and potential complications. A minimal follow-up of 3 months, corresponding to the natural delay of bone consolidation after a spine fracture, was obtained in all the cases.

### 2.5. Statistical Analysis

Student's *t*-test was performed to evaluate preoperative to postoperative changes based on radiographic measurements variables (vertebral and local kyphosis and Beck's index). For each test, the level of significance was set at 5%; that is, *P* values lower than 0.05 were considered as statistically significant.

## 3. Results

### 3.1. Population

On the whole series, mean age was 73 years (ranging from 60 to 87 years, SD 10.9). Fifteen fractures occurred in 12 patients. Etiologic distribution was severe osteoporosis in 8 cases, myeloma in 2 cases, metastasis of solid carcinoma in 1 case, and ankylosing spondylitis in the last case. The fracture occurred at L1 in 5 cases, T12 in 4 cases, T6 and T9 in 2 cases each, and T8 and T11 in 1 case.

### 3.2. Surgical Procedure

The mean procedure duration was 96.7 minutes (ranging from 85 to 110 min, SD 7.8). The instrumentation was on average performed on 5 levels (ranging from 3 to 8, SD 1.2). The screw diameter was 6.5 mm except in the 2 cases of T6 fracture in which 5.5 mm screws were inserted. During screws and rods insertion, no implant failure or pull-out was noted.

A balloon kyphoplasty was performed in 11 cases. In every case with balloon kyphoplasty, this procedure was performed after the posterior fixation in order to decrease the pressure needed to inflate the balloon and to inject the cement with low pressure to avoid leakage.

### 3.3. Radiological Outcomes

Mean preoperative vertebral and local kyphosis were 12.9° (ranging from 3 to 19, SD 5.2) and 13.2° (ranging from −4 to 27, SD 10.5),respectively. Mean postoperative vertebral and local kyphosis were 4.4° (ranging from −3 to 14, SD 4.6) and 7° (ranging from 0 to 14, SD 4.4), respectively. The difference between the pre- and postoperative vertebral kyphosis was statistically significant (12.9° versus 4.4°, *P* = 0.0006).

**Figure 3:**
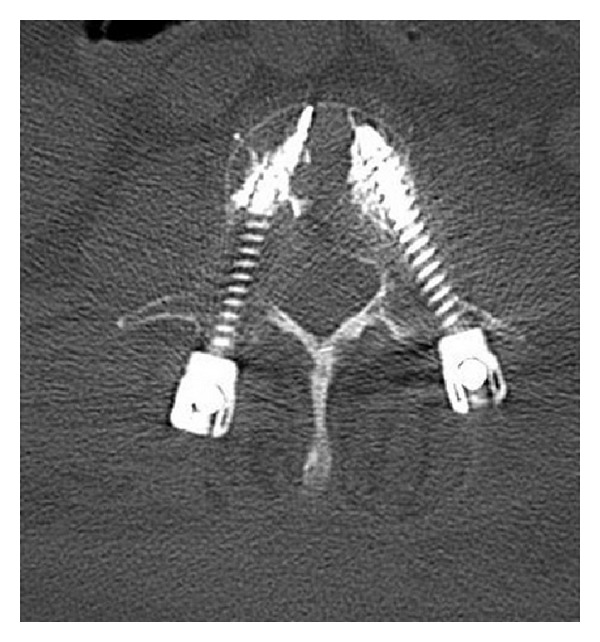
Postoperative axial CT scan showing the good positioning of the pedicular screw and the optimal placement of the screw in the vertebral body in order to inject the cement in the first anterior third of the vertebral body in order to decrease the risk of cement leakage.

Mean pre- and postoperative Beck's index were 0.57 (ranging from 0.40 to 0.71, SD 0.10) and 0.74 (ranging from 0.46 to 0.94, SD 0.14), respectively. This difference was statistically significant (0.57 versus 0.74, *P* = 0.003). Based on postoperative CT scan, and on a total of 96 screws inserted, no case of extrapedicular implant was noted (Figure 3). On the various postoperative radiological examinations, no implant failures and loosening or pull-out screws were reported at the last follow-up.

### 3.4. Clinical Outcomes

Mean length of stay was 6.4 days (ranging from 4 to 14 days). All patients used grade III analgesics before the surgical procedure. On the day of discharge, no patient used morphine. During immediate postoperative period, one patient had a pulmonary embolism due to cement leakage diagnosed on a contrast enhanced CT scan performed due to the presence of a cement leakage visible on the postoperative spine CT scan. This patient was treated with medical therapy. No other complications occurred such as infection or neurologic impairment.

## 4. Discussion

Spine surgeons are more and more concerned by aging spine and they have to deal with trauma or tumoral cases in patients with an important loss of bone stock. Performing an osteosynthesis in these patients can be difficult due to the osteoporosis and comorbidities that increase complications rates [10]. Furthermore in elderly, mechanical failures of implants and rates of pseudarthrosis are higher.

In order to decrease these operative risks, various techniques have been described. Among them, performing a percutaneous osteosynthesis can be a valuable option as it leads to a decrease of surgical time, blood loss, and infectious complications. These techniques allow a lower muscle trauma and help to a quicker postoperative recovery.

Another interest in percutaneous approach under fluoroscopic guidance is the very low rate of extrapedicular screw compared to conventional techniques [11, 12]. Using this intraoperative control, it is therefore possible to implant the screws according to the vertebral morphology in terms of length and diameter [13, 14].

However, when used alone, a percutaneous osteosynthesis can lead to a pseudarthrosis followed by screws pull-out and a recurrence of the traumatic kyphosis. In order to avoid these risks, some authors have advocated the use of long constructs. While this solution can be of interest on younger patients, we believe that in the ageing population this strategy may increase the risks of the surgical procedure. Performing an anterior support of the fractured level can therefore be necessary, using a complementary anterior approach with an intervertebral body graft or as we suggest using a balloon kyphoplasty during the same surgical session.

On the other hand, percutaneous osteosynthesis by itself is not the answer to severe osteoporosis or important loss of bone stock that can lead to screws pull-out or pedicle fracture.

The combination of percutaneous osteosynthesis with cement-augmented screws can therefore be a valuable option in the management of these fragile patients. In the past years, several biomechanical studies reported that cement-augmented screws using PMMA cement have higher pull-out strength than conventional screws [2, 15]. One of the limits of these augmented screws was related to a high risk of cement leakage associated with a nonacceptable neurologic risk. Recent developments of partially fenestrated screws are one the solutions to these risks as it allows a cement injection in the first anterior third of the screw, increasing the pull-out strength with a decreased risk of leakage [9] (Figure 3).

Recent studies reported satisfactory results of these fenestrated screws in terms of fixation strength and reduced complications [16–19].

The combination of these cement-augmented screws with a percutaneous approach seemed therefore a natural evolution for management of trauma or tumour cases in patients with poor bone stock and comorbidities.

While this study provides satisfactory clinical and radiographic results, it is crucial to respect strict rules in order to avoid complications. Each screw must be implanted in a pedicle that can accept a minimum 5.5 mm diameter screw and the length of the screw must be sufficient to reach the first anterior third of the vertebral body [13, 14]. It is also important to have a convergent approach into the vertebral body in order to reduce the risk of cement leakage. The amount of cement to be injected and its distribution into the vertebral body are also important to adapt to each case [20, 21]. A sufficient amount of cement must be injected in order to achieve a strong anchorage of the screw, but an injection of too much cement will increase the risk of leakage [22]. A maximal injection of 2 mL by screw is recommended [23] to achieve these goals and even less above T6. While between 5 and 39% of cement leakage are reported in the literature, in our experience only one patient was diagnosed with a cement pulmonary embolism related to the injection of too liquid cement.

With regard to the reduction of kyphotic deformity (mostly due to the balloon kyphoplasty) and the absence of implant failures, our results are comparable to previous series; however, this is, to date and to our best knowledge, the first series of patients treated via a percutaneous-only approach. However, further studies with a control group treated using conventional technique and a longer follow-up will be needed to confirm these results.

## 5. Conclusion

Management of severe osteoporotic thoracolumbar fractures remains a challenge for spine physicians. The use of cement-augmented screw is a valuable option for these fragile patients and can be associated with percutaneous techniques in order to be as less invasive as possible, with comparable results to conventional procedures and less morbidities.

## References

[1] Schwab F, Lafage V, Boyce R, Skalli W, Farcy J-P (2006). Gravity line analysis in adult volunteers: age-related correlation with spinal parameters, pelvic parameters, and foot position. *Spine*.

[2] Cook SD, Salkeld SL, Stanley T, Faciane A, Miller SD (2004). Biomechanical study of pedicle screw fixation in severely osteoporotic bone. *Spine Journal*.

[3] Wu Z-X, Gao M-X, Sang H-X (2012). Surgical treatment of osteoporotic thoracolumbar compressive fractures with open vertebral cement augmentation of expandable pedicle screw fixation: a biomechanical study and a 2-year follow-up of 20 patients. *Journal of Surgical Research*.

[4] Piñera AR, Duran C, Lopez B, Saez I, Correia E, Alvarez L (2011). Instrumented lumbar arthrodesis in elderly patients: prospective study using cannulated cemented pedicle screw instrumentation. *European Spine Journal*.

[5] Liu D, Wu Z-X, Pan X-M (2011). Biomechanical comparison of different techniques in primary spinal surgery in osteoporotic cadaveric lumbar vertebrae: expansive pedicle screw versus polymethylmethacrylate-augmented pedicle screw. *Archives of Orthopaedic and Trauma Surgery*.

[6] Burval DJ, McLain RF, Milks R, Inceoglu S (2007). Primary pedicle screw augmentation in osteoporotic lumbar vertebrae: biomechanical analysis of pedicle fixation strength. *Spine*.

[7] Sawakami K, Yamazaki A, Ishikawa S, Ito T, Watanabe K, Endo N (2012). Polymethylmethacrylate augmentation of pedicle screws increases the initial fixation in osteoporotic spine patients. *Journal of Spinal Disorders and Techniques*.

[8] Waits C, Burton D, McIff T (2009). Cement augmentation of pedicle screw fixation using novel cannulated cement insertion device. *Spine*.

[9] Choma TJ, Pfeiffer FM, Swope RW (2012). Pedicle screw design and cement augmentation in osteoporotic vertebrae: effects of fenestrations and cement viscosity on fixation and extraction. *Spine*.

[10] Ponnusamy KE, Iyer S, Gupta G, Khanna AJ (2011). Instrumentation of the osteoporotic spine: biomechanical and clinical considerations. *Spine Journal*.

[11] Court C, Vincent C Percutaneous fixation of thoracolumbar fractures: current concepts. *Orthopaedics & Traumatology*.

[12] Fuentes S, Blondel B, Metellus P, Gaudart J, Adetchessi T, Dufour H (2010). Percutaneous kyphoplasty and pedicle screw fixation for the management of thoraco-lumbar burst fractures. *European Spine Journal*.

[13] Heintel TM, Berglehner A, Meffert R (2013). Accuracy of percutaneous pedicle screws for thoracic and lumbar spine fractures: a prospective trial. *European Spine Journal*.

[14] Kiner DW, Wybo CD, Sterba W, Yeni YN, Bartol SW, Vaidya R (2008). Biomechanical analysis of different techniques in revision spinal instrumentation: larger diameter screws versus cement augmentation. *Spine*.

[15] Sarzier JS, Evans AJ, Cahill DW (2002). Increased pedicle screw pullout strength with vertebroplasty augmentation in osteoporotic spines. *Journal of Neurosurgery*.

[16] Barragán-Campos HM, Vallée J-N, Lo D (2006). Percutaneous vertebroplasty for spinal metastases: complications. *Radiology*.

[17] Kerry G, Ruedinger C, Steiner HH (2013). Cement embolism into the venous system after pedicle screw fixation: case report, literature review, and prevention tips. *Orthopedic Reviews*.

[18] Amendola L, Gasbarrini A, Fosco M (2011). Fenestrated pedicle screws for cement-augmented purchase in patients with bone softening: a review of 21 cases. *Journal of Orthopaedics and Traumatology*.

[19] Moon BJ, Cho BY, Choi EY, Zhang HY (2009). Polymethylmethacrylate-augmented screw fixation for stabilization of the osteoporotic spine: a three-year follow-up of 37 patients. *Journal of Korean Neurosurgical Society*.

[20] Paré PE, Chappuis JL, Rampersaud R (2011). Biomechanical evaluation of a novel fenestrated pedicle screw augmented with bone cement in osteoporotic spines. *Spine*.

[21] Hu M-H, Wu HTH, Chang M-C, Yu W-K, Wang S-T, Liu C-L (2011). Polymethylmethacrylate augmentation of the pedicle screw: the cement distribution in the vertebral body. *European Spine Journal*.

[22] Lubansu A, Rynkowski M, Abeloos L (2012). Minimally invasive spinal arthrodesis in osteoporotic population using a cannulated and fenestrated augmented screw: technical description and clinical experience. *Minimally Invasive Surgery*.

[23] Folsch C, Goost H, Figiel J (2012). Correlation of pull-out strength of cement-augmented pedicle screws with CT-volumetric measurement of cement. *Biomedical Technology*.

